# Reviews and Book Notices

**Published:** 1883-08

**Authors:** 


### Reviews and Book Notices.

ARTICLE VIII.—THE PATHOLOGY AND TREATMENT OF DISEASES OF THE OVARIES. By Lawson Tait. Fourth edition.

In this fourth edition, the author gives his peculiar views in the most pronounced manner. In the first two pages we are taught that the ovarian dermoid tumor is an analogue to the zoöspore, and that the Graaffian follicle should rather be named oögonium. Every botanist as well as every zoölogist and physiologist will energetically protest against this biological conglomerate. After having given in the next chapter the most accurate anatomica description of the ovaries and its surroundings we have ever read in any language, the anthor rides his hobby-horse i. e. menstruation. The author is well aware that there are accessory ovaries numbering sometimes from 6 to 8, and he most minutely describes the function of the fallopian tubes as oöducts, but he more than astonishes us in giving his theory, i. e. that the fallopian tubes, by encircling the ovary at the time of the ripening of the ovum, produce the phenomena called menstruation. The author peremptorily denies the similarity to menstruation of the rut in animals; and he silently admits that he has never practically observed the phenomena of repeated œstrus at distinct intervals in domesticated animals. In the next chapter the displacement of the ovaries is treated, and everybody knows that the author is always in favor of extirpation as the *sine quâ non.* His audacity in extirpating ovaries and tubes has been followed by success so far as known, but in cases of chronic suppurative inflammation he would rather abstain from operating. His ideas on extra-uterine pregnancy are properly conceived. The chapter on oöphoritis and peri-oöphoritis is classical ; andwe are sorry to find in it sentences like this : "for humanly speaking I shall always look upon you as her saviour." The author concedes in this chapter that small cystic ovaries cause hæmorrhage in the uterus. In the "conditions which simulate ovarian tumors," the author repeates his biological reasonings in the above named characteristic manner, but there are many points of interest and truth. The practical part of this and the next chapter on ovariatomy, forms the basis of the book. There the author shows his thorough knowledge of topographical anatomy and his large surgical experience. There is a minute description of all the essential points in this widely executed operation. American priority in ovariotomy and oöphorectomy is denied, but due credit given to McDowell and Battey. The last chapter on "recent extensions of abdominal tumors," is rather eccentric, the author favoring exploration of the pelvic cavity in all cases of abdominal diseases not due to malignant growths, and he gives the characteristic opinion that "risk of life in chronic ill-health is no argument against operation." Pelvic suppuration, as the author calls it, is treated by abdominal section and drainage, but the author's experience on this point seems to be rather short and not successful. The book has a fine exterior and the print is large and distinct. The author should attend to the correct printing of the German book-titles. The book has so many interesting points that even the most scrupulous reader will lay it aside with entire satisfaction. O. S.

ARTICLE IX. — FIRST AID TO THE INJURED. By PETER SHEPHERD, M.D., Surgeon-Major, Army Medical Department; Associate of the Order of St. John of Jerusalem. Revised by Bowdich Morton, M.D. Cloth, pp. 88. New York: G. P. Putnam's Sons. Chicago, Jansen, McClurg & Co.

This little work is gotten up in nearly the same style as the Student's Aid Series. The author states in the preface, that there is no attempt made to popularize medicine and surgery; the object is to furnish a few plain rules, which may enable any one to act in cases of injury or sudden illness, pending the arrival of professional help.

The professsonal help called would sometimes be able to treat such cases more intelligently, if familiar with the contents of this book.

The first part of the book is devoted to an outline of anatomy and physiology.

In speaking of the ribs, the statement is made that the lower five are termed floating or false ribs, having no attachment in front. We believe the two lower ribs only are, properly speaking, termed floating ribs, "having no attachment in front." The three just above the floating ribs are attached in front to the true ribs by means of cartilage. The lower five are false ribs. The membrana tympani is called the drum of the ear. The membrana tympani is the head of the drum, tympanum, or middle ear, but is not itself alone the drum or middle ear.

With these exceptions, the book is very correct. The latter half of the work contains much valuable matter, which might be read over many times with advantage to the reader.

J. T. M.

ARTICLE X.—POTT'S DISEASE: ITS PATHOLOGY AND MECHANICAL TREATMENT, with Remarks on Rotary Lateral Curvature. By NEWTON M. SHAFFER, M.D., Surgeon in Charge of the New York Orthopœdic Dispensary; Orthopœdic Surgeon to St. Luke's Hospital, New York. Cloth, pp. 82. New York: G. P. Putnam's Sons. Chicago: Jansen, McClurg & Co. Price $1.00.

This is a plain, concise, and practical work on Pott's Disease. The author gives the classes, causes, and symptoms of the disease, in a clear style. The treatment is based on the pathology, which is prominently considered. Attention is often called to the similarity of Pott's disease and osteitis of the hip- or knee-joint. Hence the same rule should govern the treatment in both.

The author has been treating his patients having Pott's disease with an antero-posterior support, using a chin-piece to steady the head, when the disease is in the cervical or upper dorsal regions. The advantages of this over the plaster jacket are many, namely: it is lighter, and more comfortable to the wearer; it is easier adjusted; there is no need of suspension in application; pressure is applied just where it is needed most; respiration and transpiration are not interfered with, and cleanliness of the body is better secured.

Surgeons who are still treating Pott's Disease with the plaster jacket should read this book, and give the antero-posterior support a trial. We think if they would do so, the plaster jacket would be soon discarded entirely in the treatment of this disease.

J. T. M.

ARTICLE XI.—TREATMENT OF VARICOCELE BY EXCISION OF REDUNDANT SCROTUM. Illustrated by New Instruments and an Account of Fifteen Successful Cases. By M. H. HENRY, A.M., M.D., Late Surgeon-in-Chief to the State Emigrant Hospitals, Ward's Island, New York; Fellow of the New York Academy of Medicine, etc., etc. Cloth, pp. 24. New York: J. H. Vail & Co.

Of all operations for the radical cure of varicocele, the one described by the author of this little book, experience shows, is the safest and best.

The author has devised a clamp or scrotal forceps to be used in this operation. A fold of the scrotum is held firmly by the instrument during the operation. As much of this fold as is thought necessary is removed with the knife or scissors. The clamp keeps the edges of the wound in place, and is not removed till sutures are inserted.

By using the clamp, not only can the operation be done very quickly, as the testicles do not become exposed, or interfere with the introduction of sutures, but the author claims he can get union by first intention in the majority of cases. Thus the instrument appears to be a very useful one, especially would it be so to a surgeon who often performs this operation.

The pathology of varicocele receives its share of attention. Members of the profession to whom this operation is new would do well to peruse this work.

J. T. M.

### Books and Pamphlets Received.

### BOOKS.

Variola. Samuel A. Powers.

Diseases of the Ovaries. Lawson Tait. Wm. Wood & Co. W. T. Keener.

Diseases of Women. Frisch. Wm. Wood & Co. W. T. Keener.

Diagnosis of Ovarian Cysts by Examination of their Contents. Garrigues. Wm. Wood & Co. W. T. Keener.

Report Michigan State Board of Health, Lansing, Mich.

Illinois State Medical Society Report, 1882.

Medical and Surgical History of the War of the Rebellion. Part Third. Vol. Second. Surgical History. First issue.

Therapeutic Handbook of the Pharmacopœia. Wm. Wood & Co. W. T. Keener.

The Microscope and Its Revelations. Carpenter. Wm. Wood & Co. W. T. Keener.

How to Examine the Chest. Samuel West. Philadelphia, Blakeston's Son & Co. Chicago, W. T. Keener.

The American Citizen's Manual. W. C. Ford. N. Y., G. P. Putnam's Sons.

Alcoholic Inebriety from a Medical Standpoint. Joseph Parish. Phila., P. Blakeston's Son & Co.

Aids to Medicine. C. Armand Semple. N. Y., G. P. Putnam's sons.

First Annual Report of the Provincial Board of Health of Ontario, Year 1882. Toronto, C. B. Robinson.

Treatise on Insanity in its Medical Relations. Wm. A. Hammond. N. Y., D. Appleton & Co. Jansen, McClurg & Co.

Insanity: Its Causes and Prevention. Henry Putnam's Sons. N. Y., G. P. Putnam's sons. Junsen McClurg & Co.

Diseases of the Throat. Carl Seiler. Phila., H. C. Lea's Son & Co. Jansen, McClurg & Co.

Taylor's Medical Jurisprudence. Third edition. Vols. I. and II. Phila., H. C. Lea's Son & Co. Jansen, McClurg & Co.

Biddle's Materia Medica. Ninth edition. P. Blackston, Son & Co. W. T. Keener.

Practitioner's Ready Reference Book. R. J. Dunglison. Phila., Blakeston, Son & Co. W. T. Keener.

Bacteria and the Germ Theory of Disease. H. Gradle. W. T. Keener.

Relations of Micro-organisms to Disease. W. T. Belfield. N. Y., Wm. Wood & Co. W. T. Keener.

Examination of Modern Chemicals. Hoffman & Power. Phila., H. C. Lea's Son & Co. Jansen, McClurg & Co.

Diseases of the Male Sexual Organs. Samuel W. Gross. Henry C. Lea's Son & Co.

Diseases of the Eye. Second edition. Edw. Nettleship. Henry C. Lea's Son & Co. Jansen, McClurg & Co.

In-Knee and Its Relation to Rickets. W. J. Little. N. Y., D. Appleton & Co. Jansen, McClurg & Co.

Lectures on Medical Nursing. J. W. Anderson. N. Y., MacMillan & Co. W. T. Keener.

Ausculation and Percussion. Manual of Austin Flint. Third edition. Phila., Henry C. Lea's Son & Co. Jansen, McClurg & Co.

Handbook of Electro-Therapeutics. Wilhelm Erb. Trans. by Putzel. N. Y., Wm. Wood & Co. W. T. Keener.

International Encyclopædia of Surgery. Vol. III. N. Y., Wm. Wood & Co. W. T. Keener.

PAMPHLETS.

Clinical Lecture on the Treatment of Caries of the Lumber Vertebræ. M. J. Roberts.

Ergotin and Pelletierine. Ch. Tauret.

History of Health Work in Indiana. Thad. M. Stevens.

What is Disease. W. Godfrey Dyas.

Clinical Notes on Opium Addiction. J. B. Matteson.

Cough of Remote Origin. W. E. Cassellbery.

Homicide and Suicide in Philadelphia, 1871 to 1881. Jno. G. Lee.

Hints on the Treatment of Some Parasitic Skin Diseases. Geo H Rohé.

Treatment of Various Forms of Acne. Geo. H. Rohé.

Pemphygus and Some Diseases Liable to be Mistaken for It. G. H. Rohé.

The Prevalence af Pneumonia in Chicago in 1882. N. S. Davis.

General Paralysis of the Insane. T. F. McFarland.

The Hypodermic Treatment of Syphilis. A. Lagorio.

The Treatment of Bubo. G. Frank Lydston.

